# Supplementary material for: Genome-Wide Analysis of the HDAC Gene Family and Its Functional Characterization at Low Temperatures in Tartary Buckwheat (Fagopyrum tataricum)
Source: Int J Mol Sci. 2022 Jul 10;23(14):7622. doi: 10.3390/ijms23147622 (PMC9319316; doi:10.3390/ijms23147622)
Supplement: Supplementary file 1 [file ijms-23-07622-s001.zip › supplemental material-Figures.pdf]

Genome-wide analysis of the HDAC gene family and its functional characterization at low temperatures in Tartary buckwheat (*Fagopyrum tataricum*)

supplemental figures

Yukang Hou[1,†], Qi Lu[1,†], Jianxun Su[1], Xing Jin[1], Changfu Jia[2], Lizhe An[1], Yongke Tian[1,*] and Yuan Song[1,*]

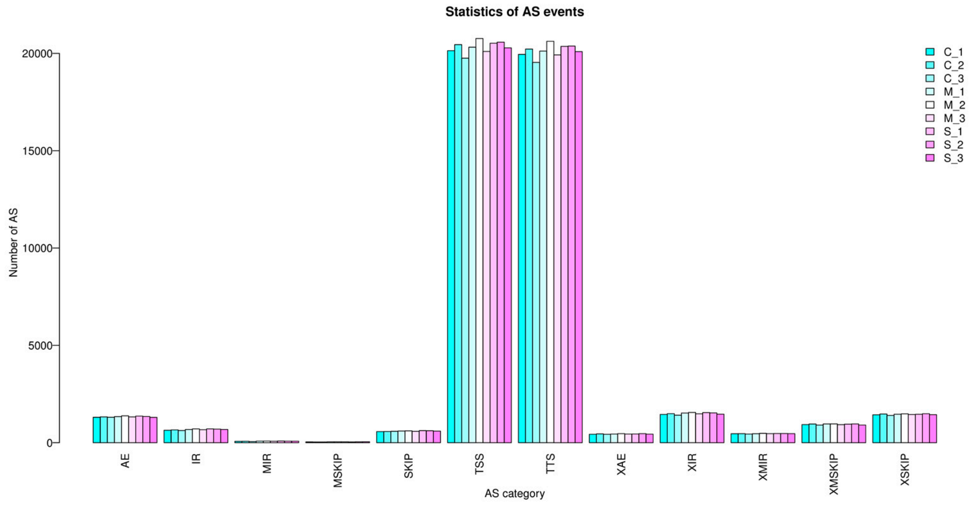

Figure S1. statistics of alternative splicing of Tartary buckwheat. The main variable shear events are as follows: Exon skipping (SKIP), cassette exons (MSKIP): single exon skipping, multi-exon skipping; retention of single (IR), multiple (MIR) introns: single intron retention, multiple intron retention; alternative exon ends (AE): Variable 5 ′ or 3 ′ shear; alternative transcription start site (TSS): variable first exon shear; alternative transcription termination site (TTS): variable shear of the last exon. Five other fuzzy boundary variable shear, as follows: Approximate SKIP (XSKIP): single exon jump (fuzzy boundary); Approximate MSKIP (XMKIP): Multi-exon skipping (fuzzy boundary); Approximate IR (XIR): single-intron retention (fuzzy boundary); Approximate MIR (XMIR): multi-intron retention (fuzzy boundary); Approximate AE (XAE): Variable 5 'or 3' shear (AE) (fuzzy boundary).

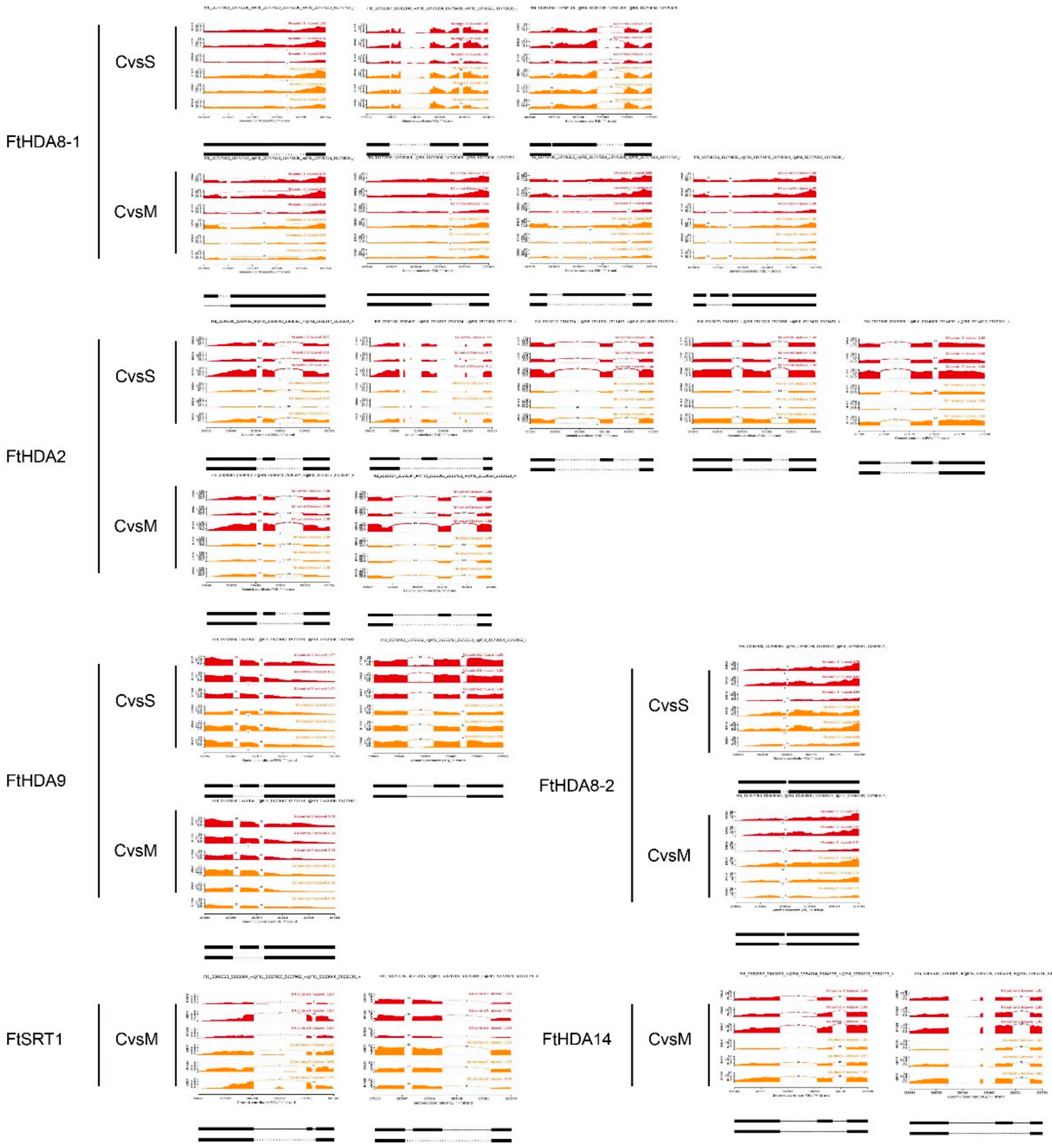

Figure S2. Alternative splicing events of *FtHDACs* are associated with different low temperature treatment. Sashimi plot indicating the average RNA-seq read density and splice junction counts for each genotype. Exons and introns are not drawn to scale, and the relative width of exons is increased for clarity. C: living at room temperature always; S: not acclimated, directly exposed to 0°C for 6 h; M: 4°C for 6 h, then at room temperature for 18 h, repeated four times, and then placed at 0°C for 6 h. C group (red) served as a negative control for S/M group (orange) enrichment.

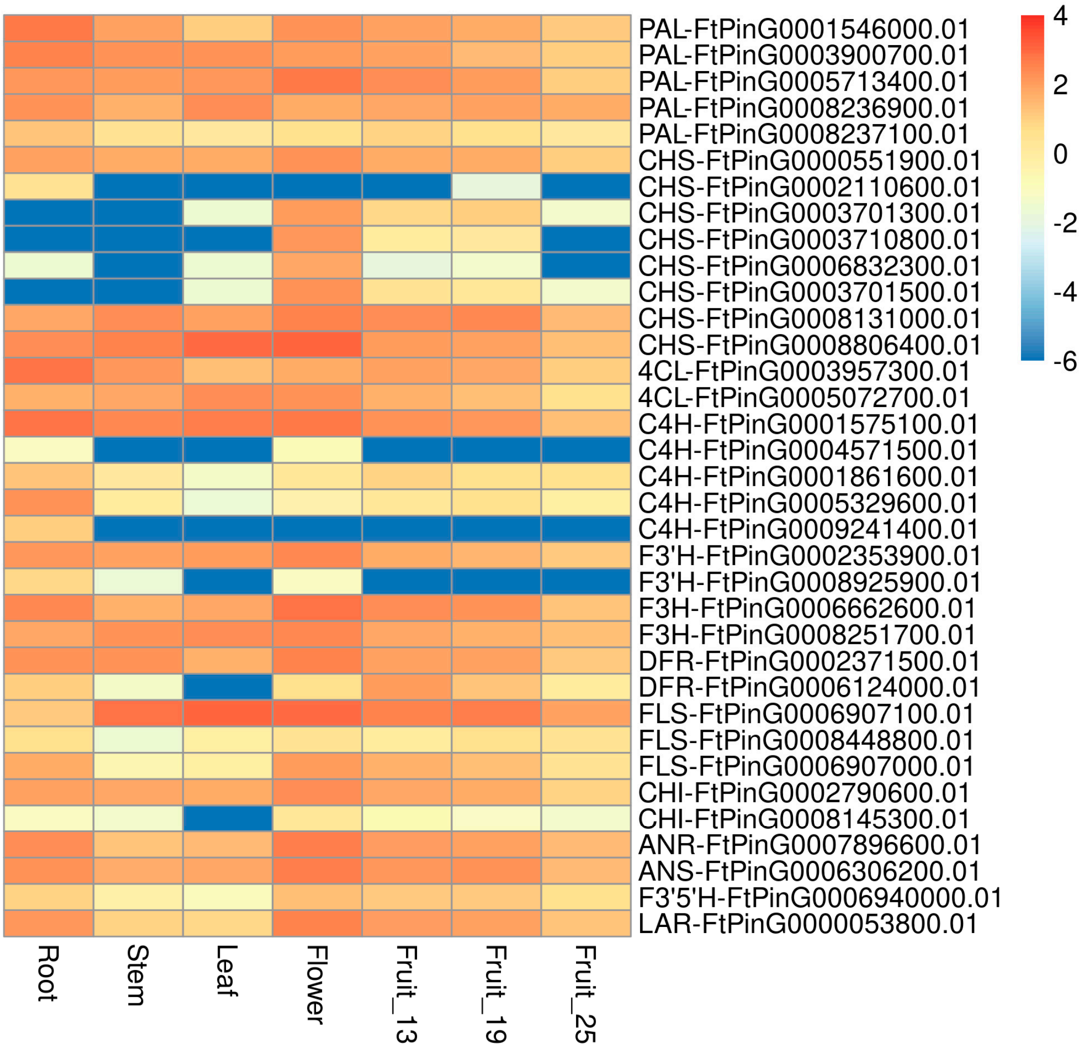

Figure S3. Expression patterns of some flavonoid synthesis genes in various Tartary buckwheat tissues. RNA-seq expression data were retrieved from Tartary Buckwheat Database (TBD) for further analysis. The expression in various Tartary buckwheat tissues was shown, including root, stem, leaf, flower, and fruit_13, fruit_19, fruit_25. Blue to red, high to low in the expressional level.

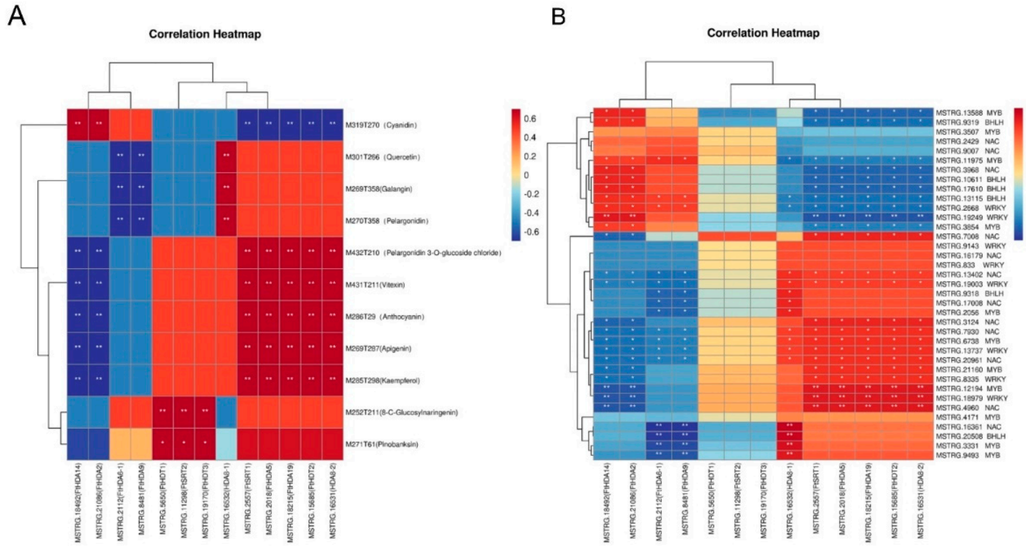

Figure S4. The correlation analysis of *FtHDACs* and (A) metabolite accumulation or (B) TFs genes expression. The data was extracted form transcriptome and metabolome of the three cold test groups: control group (C), cold memory group (M), and cold shock (S). Red indicates the positive correlation, and blue indicates a negative correlation. Small white stars indicate significant associations (⋆: $P \leq 0.05$， ⋆⋆: $P \leq 0.01$).

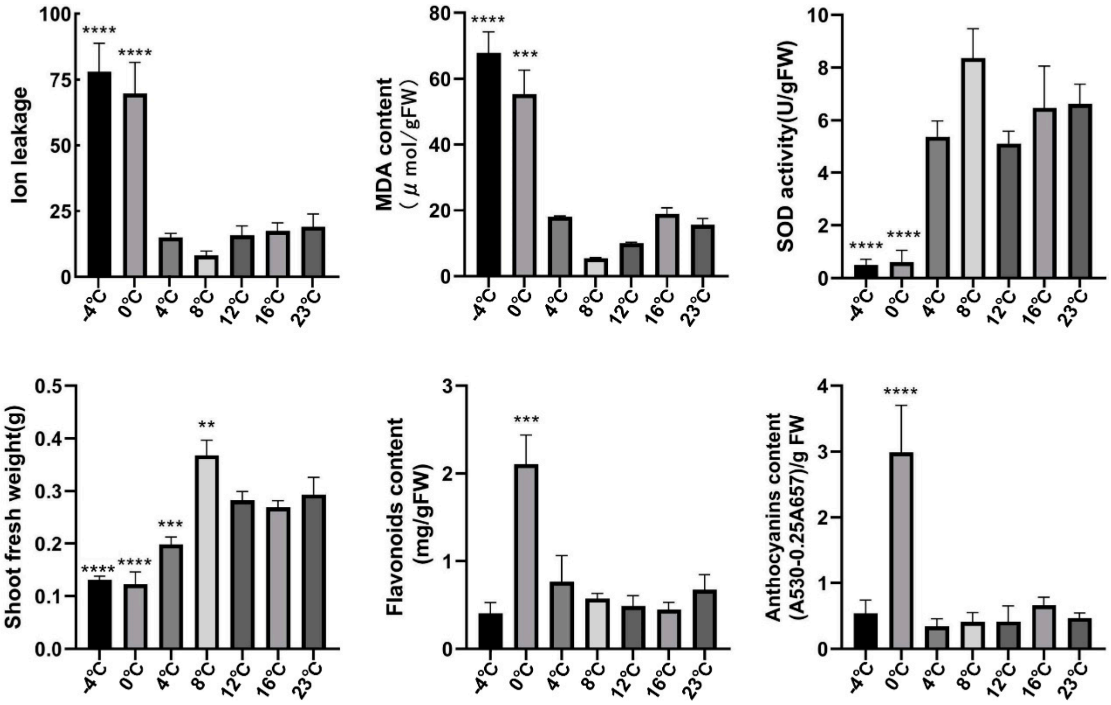

Figure S5. Measurement of the freezing resistance of Dingku1. Electronic leakage rate, Malondialdehyde (MDA) content, SOD, shoot fresh weight, flavonoids and anthocyanins were tested.

The mean value was from more than 30 independent plant measurements, and error bars indicated ± SD. Analysis was performed with one-way ANOVA (and nonparametric or mixed), ****P<0. 0001.

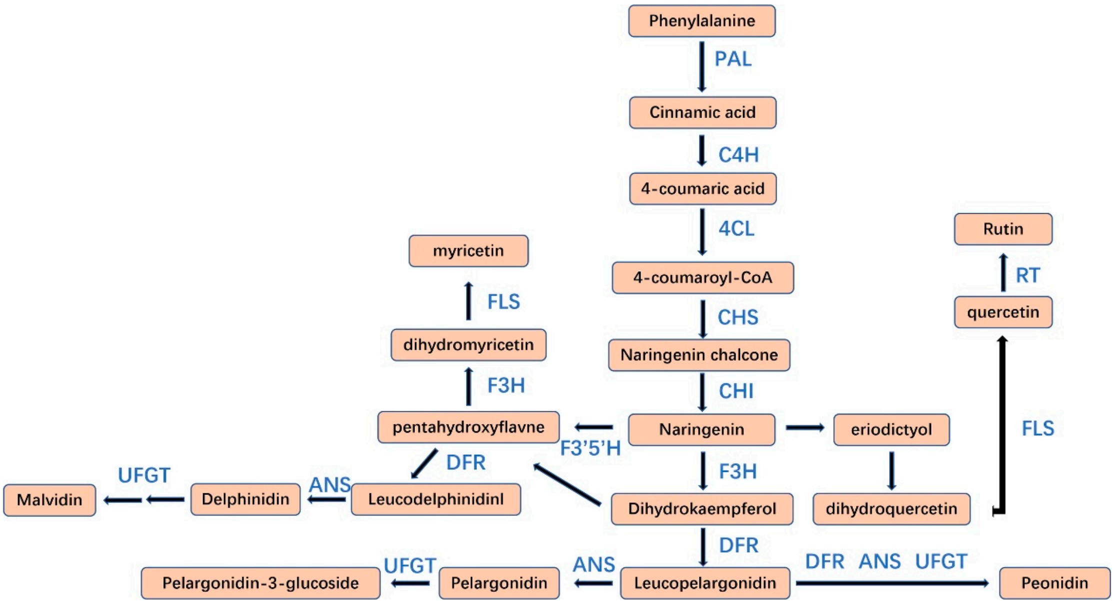

Figure S6. Enzymes on the flavonoid synthesis pathway.

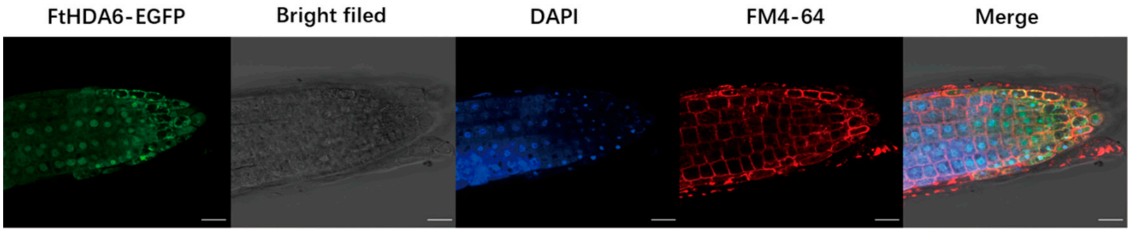

Figure S7. Fluorescent identification of FtHDA6-EGFP in transgenic lines. Bars=20μm

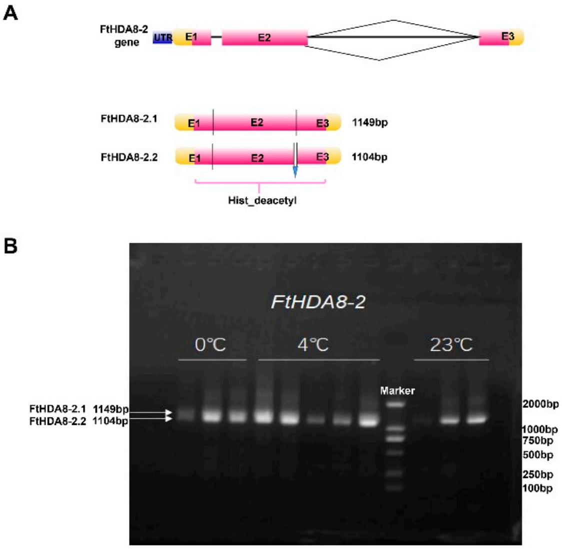

Figure S8. Alternative splicing of FtHDA8-2. (A) gene structure of FtHDA8-2 and the alternative splicing pattern. Alternative 5′ splice sites in the second intron generate alternative splicing isoforms of FtHDA8-2.1 and FtHDA8-2.2 transcripts. (B) PCR analysis using *FtHDA8-2* ORF primers and cDNA template from Tartary buckwheat 3-weeks seedlings treated at 0 °C, 4 °C for 6h and 23 °C (the control). Boxes represent exons, lines represent introns, and diagonal lines represent splicing event.
